# Urology Residents’ Perspectives on the In-House On-Call Systems: A Study in an Accredited Academic Center

**DOI:** 10.5339/qmj.2022.49

**Published:** 2022-11-09

**Authors:** Ibrahim A. Khalil, Tarek Ibrahim, Maya Aldeeb, Ahmed Mohamed, Rym Ben Salah, Omar M. Aboumarzouk, Abdulla Al-Naimi

**Affiliations:** ^1^Department of Urology, Hamad medical corporation, Doha, Qatar E-mail: Ikhalil1@hamad.qa; ^2^Department of Medical Education, Family Medicine Residency Program, Hamad Medical Corporation, Doha, Qatar; ^3^Department of Surgery, Surgical Research Section, Hamad Medical Hospital, Hamad Medical Corporation, Doha, Qatar; ^4^College of Medicine, Qatar University, Doha, Qatar; ^5^The College of Medical, Veterinary and Life Science, University of Glasgow, Scotland, UK

**Keywords:** Surgical education, quality of life, urology Residency program, on-call systems, 12-hour on-call system, postgraduate training

## Abstract

Introduction: Medical education and training are crucial in maintaining patients’ safety and improving patient care quality. Multiple studies have evaluated the effects of restrictive policies on the resident's quality of life and education. Due to the compiling data and the fact that these trials evaluated programs with a substantial number of residents, it remains uncertain whether these conclusions can be extended to urology programs with a small number of residents. Multiple on-call systems have been adopted in residency programs across the world. This study evaluated the residents’ quality of life, clinical experience, and education upon transitioning from 24-hour to 12-hour in-house on-call systems.

Methods and materials: In this observational and questionnaire-based study, the effect of the transition from 24-hour to 12-hour in-house on-call systems was compared in terms of the resident's quality of life and education, surgical case volume, and working hours’ rules compliance.

*Quality of life and education*: We adopted a validated survey based on a 5-point Likert scale to assess the residents’ perception of the transition to a 12-hour on-call system on their quality of life and education.

*Surgical case volume*: We extracted the number of cases the residents operated on from the operating theater database at our institution.

*Working hours: compliance and violations*: The weekly working hours, compliance, and violations per ACGME-I rules were collected from the MedHub platform.

Results: *Quality of life and education:* Residents rated the 12-hour on-call system superior in terms of quality of life, education, and surgical case volume.

*Surgical case volume*: There was a 45% increment in the surgical case volume (*p* = 0.04) with the 12-hour on-call system.

Working hours: compliance and violations

There was no significant difference in the mean weekly working hours (*p* = 0.1). However, the total number of duty hours violations decreased in the 12-hour on-call system.

Conclusion: The 12-hour system is a better alternative to the 24-hour system in terms of the resident's quality of life, education, surgical case volume, and compliance with duty hour rules.

## Introduction

Medical education and training are crucial in maintaining patients’ safety and improving patient care quality. Therefore, residency programs are recommended to follow quality standards that ensure the grooming of highly qualified professional physicians and surgeons who can deliver safe, high-quality, evidence-based medical care to patients. Hamad Medical Corporation (HMC) is the leading governmental organization in Qatar, with more than 14 hospitals that provide medical care and postgraduate training in Qatar and are committed to delivering the safest and most effective patient care.^1–3^ HMC was one of the first institutions to achieve Accreditation Council for Graduate Medical Education-International (ACGME-I) accreditation in the Middle East, an independent, not-for-profit, physician-led organization that sets and monitors professional educational standards.^4^

ACGME-I established the shift length limits in 2011, after which different studies evaluated the effects of restrictive policies on the resident's quality of life (QoL) and education. The individualized Comparative Effectiveness of Models Optimizing Patient Safety and Resident Education (iCOMPARE) trial found that restrictive policies are associated with higher residents’ satisfaction with their education and overall well-being.^5,6^ On the other hand, the Flexibility in Duty Hour Requirements for Surgical Trainees trial found no change in the resident satisfaction quotient with the application of restrictive duty hour policies.^7^ Due to the compiling data and the fact that these trials evaluated programs with a substantial number of residents, the extension of these conclusions to a urology residency program with a small number of residents remains uncertain.

The Urology Residency Program at HMC has been accredited by the ACGME-I since 2012.^1^ We used to adopt the 24-hour in-house on-call system, which is tedious and inconvenient to the residents, especially after a recent hospital expansion and working across multiple sites.^8^ These new changes and requirements necessitated changes to the on-call system in order to accommodate the expansion in services while maintaining the residents’ compliance with the standards of ACGME-I.

Multiple on-call systems have been adopted in residency programs worldwide, including the in-home or in-house 24-hour, morning duties with a night float, and 12-hours on-call systems.^9,10^ To the best of our knowledge, there is only one study that evaluated the on-call system effect in urology programs. In their study, Mohapatra et al. included 19 urology residents covering five academic hospitals and found that the transition from home call to night float system improved the residents’ QoL and duty hours compliance, with no effect on the resident surgical case volume.^11^ However, no evidence supports one in-house call system over the other.

This study aimed to evaluate the residents’ QoL, clinical experience, and education upon transitioning from 24-hour to 12-hour in-house on-call systems.

## Methods And Materials

In this observational and questionnaire-based study, we evaluated the transition of the urology residency program's on-call system at HMC in 2020. All urology residents working at HMC during the pre-and post-change phases in this study were included (n = 8). The small sample size included in our study can be attributed to the fact that urology residency programs worldwide have a limited number of residents, which is the same for our program. We examined the effect of on-call system change on residents’ QoL, education, surgical case volume, and working hours rules’ compliance.

The methodology was reviewed and approved by the Surgical Institution Board Review of HMC. All procedures were conducted in accordance with the established ethical guidelines, with assured confidentiality, and informed consent was obtained from all participants.

### QoL and education

We adopted a validated survey based on a 5-point Likert scale.^12,13^ To assess the residents’ perception of the transition to a 12-hour on-call system on their QoL and education (Appendix 1), where 1 = strongly disagree and poor effect and 5 = strongly agree and positive effect on the specific measured domain. We used the sections that cover our study objectives because the original questionnaire has multiple domains not included in our study. All participants completed the survey anonymously 1 year after the transition to the 12-hour on-call system.

### Surgical case volume

The evaluation of the surgical case volume was performed from April to July 2019 for the pre-change and then in the same period in 2021 for the post-change phase. Although the transition occurred in December 2020, the period mentioned above was selected to avoid the restriction measures applied to elective surgeries during the COVID-19 pandemic.^14^ We extracted the number of cases the residents operated on from the operating theater database of our institution.

### Working hours: compliance and violations

The weekly working hours, compliance, and violations as per the ACGME-I rules were collected from the MedHub platform which is a software used by the residency programs to monitor the workflow of the residents.^15^ We monitored the rule of a maximum of 80 working hours/week, a maximum of 24+4 hours of continuous duty, and at least one free day every week.

### Statistical analysis

We calculated the duty hours, the surgical case volume, and the total number of violations for all residents during both phases of the study. Paired t-sample tests were performed to compare the percentages of the residents’ cases and the means of the weekly working hours in pre and post-change phases. *p* < 0.05 was considered to indicate statistical significance.

## Results

### QoL and education

**Figure 1.**
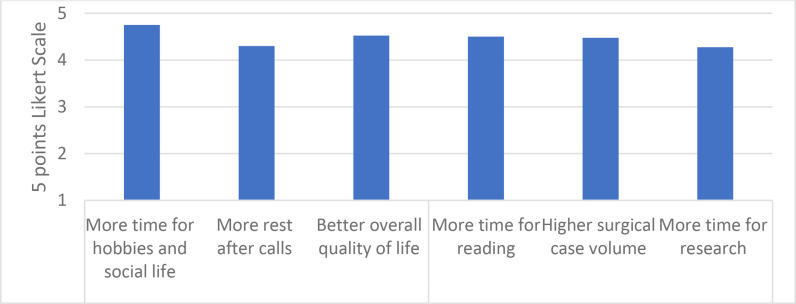
Mean survey response for resident (5 points Likert scale), where 1 =  strongly disagree and 5 =  strongly agree

All eight urology residents completed the survey, giving a response rate of 100%. Residents rated the 12-hour on-call system superior in all tested parameters, namely, QoL, education, and surgical case volume, with a high Likert scale score >4 [mean = 4.5, S.D = 0.68]. Residents’ perceptions of the effect of transition on their QoL and education are presented in (Figure 1).

### Surgical case volume

**Figure 2.**
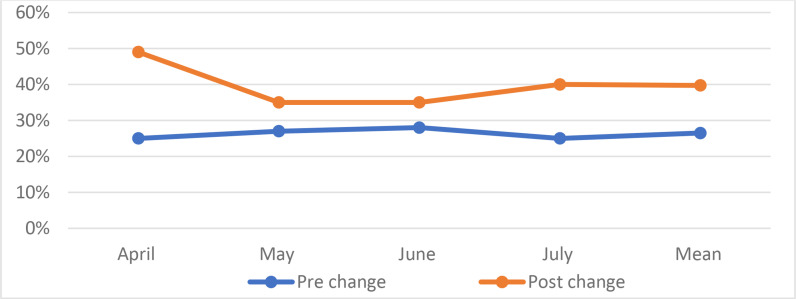
Percentages of cases operated by residents

In the pre-change phase, the residents were involved in 188 out of 694 (27%) cases, while in the post-change phase, they were involved in 262 out of 674 (39%) cases. This change corresponded to a 45% increment in the surgical case volume (*p* = 0.04). The complexity of cases in both phases is comparable. The percentages of cases operated by the residents are presented in Figure 2.

### Working hours: compliance and violations

**Figure 3.**
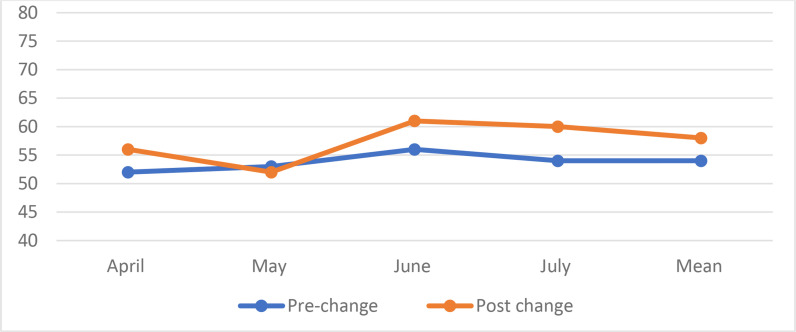
Working hours per resdient per week

**Figure 4.**
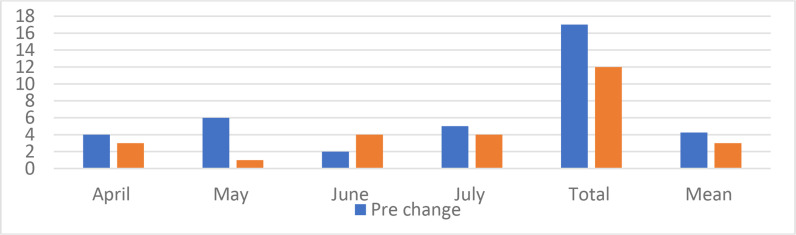
Working Hours rules violations per month

No statistically significant difference was noted in the mean weekly working hours; 54 and 58 hours for pre and post-change phases, respectively (*p* = 0.1). However, the total number of duty hours violations decreased from 17 to 13 after the change, with a relative decrease of 24%. The weekly working hours and violations are plotted in Figures 3 and 4.

## Discussion

Different studies evaluated the effect of the on-call system on resident education and QoL, but most of these studies were conducted in programs with large sample sizes, unlike in the urology residency programs, which involve a small number of residents. This study assessed the impact of the transition of an in-house on-call system from 24 hours to 12 hours on the QoL, clinical experience, and education of urology residents. We found that implementing the 12-hour on-call system improved their QoL and led to an increase in the number of surgical cases they handled without affecting their average working hours.

Decreased QoL scores among the residents during their training years were associated with several drawbacks, including an unhealthy lifestyle, psychological problems, academic failure, and negative impacts on their professional development.^16–21^ Afana et al. found that the most frequent stressors in HMC residency programs were related to working hours and workload.^22^ In their study, Mohapatra et al. demonstrated that the night float system improved the residents’ QoL when compared to that with home calls.^11^ Other studies support that compliance with ACGME duty hours rules achieved a better overall resident QoL.^23^ Our study found that the 12-hour on-call system improved the QoL for urology residents, where they found more time for social life, hobbies, reading, and research activities. Residents also reported better rest after the on-call duties. We attribute this report to the shorter shift times and longer rest times and to the flexibility of the system to adapt to the need of residents, which added more value to its effect on QoL. In the 12-hour on-call system, the resident has 12 hours of duty, followed by 12 hours of rest in the morning shift, while, in the night shift, the residents cover a maximum of 20 hours of duty, followed by at least 16 hours of rest.

Surgical training is predominantly action-based^24^; increasing surgical case volume has been associated with improved performance and increased trainees’ confidence.^25,26^ Comparing the night float system, in which dedicated residents cover nights and are off duty during the day, to the in-home call, Mohapatra et al. found no difference in the surgical case volume handled.^11^ In contrast, Scott et al. found that a 24-hours on-call system was associated with better residents’ surgical experience than a night float system (27). Our study found an increase in the surgical case volume favoring the 12-hour system. This difference may be attributed to the fact that our on-call system is in-house rather than in-home calls. The flexibility of the 12-hour on-call system, which avoids overlap between operating theater days and calls, and fewer working hours’ violations led to an enhanced overall surgical experience.

Compliance with ACGME-I working hours rules^14^ had positive effects on the residents’ lifestyle, satisfaction level with clinical learning, and education without any adverse effects on their operative experience.^28–31^. Adherence to the ACGME-I rules among HMC residents has previously been associated with improved examination and research performance.^32,33^ The same finding was recorded in the present study, as residents reported more time for studying, exam preparations, and research activities with the 12-hour on-call system. On the other hand, Nevin et al. found that the implementation of the AGMCE duty hours rule was associated with fewer hours spent in the hospital, which inevitably decreased hands-on clinical education.^34^ In our study, there was no change in the average weekly working hours per resident and fewer working hours violations in the 12-hour on-call system. Reduction in the working hour violations without affecting the average working hours per week led to improved QoL, education, research, and surgical experience among the urology residents. We attribute this observation to the flexibility of the 12-hour on-call system and the limitation imposed on the maximum possible continuous working hours to 20 hours with this system.

Regarding the effect of ACGME duty hours limitations^14^ on patient safety and QoL provided by residents, several studies have reported that adherence to ACGME rule is associated with improved patient safety,^35–37^ while others reported no change or even decreased patient safety.^38–40^ McGahan et al. found that patient-related outcomes are comparable between the 24-hour on-call system and the night float system,^41^ while Mohapatra et al. reported that the night float system is associated with better overall patient care, which could be attributed to the shorter continuous duties.^11^ Although the finding of better patient care found in the night float systems can be expanded to a 12-hour on-call system due to the similar duties duration. The effect of a 12-hour on-call system on patient care and safety compared to other systems warrants further studies.

This study has some limitations. Although all urology residents who were involved in the study completed the survey, their number was limited to establish any solid evidence. Moreover, the study duration should have exceeded six months period. Furthermore, we believe that the emerging situation of the COVID pandemic, along with its effects on surgical training, would have affected our results. We preferred to perform our study to evaluate the on-call systems during normal circumstances without any restrictions. Another limitation of this study is that the effect of on-call system change on patient safety was not addressed.

Despite these limitations, our study presents the first evidence demonstrating the benefits of applying 12-hour rather than 24-hour in-house on-call systems for urology residents.

## Conclusion

For urology residents who cover in-house on-call duties, the 12-hour system is a better alternative to the 24-hour system in terms of residents’ QoL, education, surgical case volume, and compliance with the duty hour rules. Further multi-center studies are warranted to evaluate other on-call systems that involve a larger number of urology residents and their effect on resident education and QoL and to evaluate whether there is a difference among different on-call systems.

### Conflicts of interest

The authors have no conflicts of interest to declare that they are relevant to the content of this article.

### Acknowledgment

We thank Mrs. Annu Kattikaren, the Urology Resident Program coordinator at HMC, for her help in the retrieval of data used in this manuscript.

## Figures and Tables

**Table:** 

Strongly disagree	disagree	Neither agree nor disagree	Agree	Strongly agree


**Table:** 

Strongly disagree	disagree	Neither agree nor disagree	Agree	Strongly agree


**Table:** 

Strongly disagree	disagree	Neither agree nor disagree	Agree	Strongly agree


**Table:** 

Strongly disagree	disagree	Neither agree nor disagree	Agree	Strongly agree


**Table:** 

Strongly disagree	disagree	Neither agree nor disagree	Agree	Strongly agree


**Table:** 

Strongly disagree	disagree	Neither agree nor disagree	Agree	Strongly agree


## Appendix 1

**Residents’ Quality of Life and Education Survey**

**A. Quality of life:**

1. After the transition to the 12-hour on-call system, I have more time for my hobbies and social life

2. After the transition to the 12-hour on-call system, I have more rest after calls

3. After the transition to the 12-hour on-call system, I have an overall better quality of life

**B. Residents’ education and clinical experience**

1. After the transition to the 12-hour on-call system, I have more time for reading (studying and reviewing literature and guidelines)

2. After the transition to the 12-hour on-call system, I have a higher surgical case volume

3. After the transition to a 12-hour on-call system, I have more time for research activities
